# Predictive Value of Red Blood Cell Distribution Width in Chronic Obstructive Pulmonary Disease Patients with Pulmonary Embolism

**DOI:** 10.1155/2020/1935742

**Published:** 2020-07-21

**Authors:** Jing Wang, Zongren Wan, Qing Liu, Baolan Wang, Liang Wang, Dan Yang, Lixin Wang, Yongqing Hong, Rong Zhu

**Affiliations:** Department of Respiratory Medicine, The Huaian Clinical College of Xuzhou Medical University, Huaian 223001, China

## Abstract

**Purpose:**

This study is aimed at investigating the relationship between red cell distribution width (RDW) and chronic obstructive pulmonary disease (COPD) patients with pulmonary embolism (PE).

**Methods:**

We conducted a retrospective study enrolling a total of 125 patients from January 2013 to December 2019. The study group consisted of 40 COPD patients with PE, and the control group had 85 COPD patients without PE. Clinical data including demographic characteristics, comorbidities, and results of imaging examinations and laboratory tests were recorded. Blood biomarkers, including red blood cell distribution width standard deviation (RDW-SD), red blood cell distribution width coefficient of variation (RDW-CV), and D-Dimer, were included.

**Results:**

RDW-SD and RDW-CV were higher in the COPD patients with the PE group (*p* < 0.001). A higher RDW-SD led to a significantly increased risk of PE than a lower RDW-SD (adjusted odds ratio (OR): 1.188; 95% confidence interval (CI): 1.048-1.348). The area under the curve (AUC) of RDW-SD used for predicting PE was 0.737. Using 44.55 as the cutoff value of RDW-SD, the sensitivity was 80% and the specificity was 64.7%. The prediction accuracy of RDW-SD combined with D-Dimer (AUC = 0.897) was higher than that of RDW-SD or D-Dimer alone. The optimal cutoff value of RDW-SD+D-Dimer for predicting PE was 0.266, which generated a sensitivity of 87.5% and specificity of 83.5%.

**Conclusion:**

RDW is significantly increased in COPD patients with PE and may thus be useful in predicting the occurrence of PE in patients with COPD.

## 1. Introduction

Pulmonary embolism (PE) is a clinical manifestation of venous thromboembolism (VTE), in which flowing emboli block pulmonary arteries and ultimately leads to sudden death. VTE is a major contributor to the global disease burden and poses a major public health challenge [1]. The Global Burden of Disease Study estimated that thromboembolic conditions accounted for one in four deaths worldwide in 2010 and are a leading cause of mortality [2]. In epidemiological studies, annual incidence estimates for PE increased from 85 per 100,000 population in 2005 to 109 per 100,000 population in 2015, ranking the third among the causes of cardiovascular mortality [3].

Chronic obstructive pulmonary disease (COPD) is a chronic inflammation of the respiratory tract and lungs that reduces airflow and causes damage to lung tissue. The Global Burden of Disease Study estimated that COPD will be the third leading cause of death in the world by 2030 [4]. Moreover, COPD has recently been recognized as an independent risk factor for PE [5]. A population-based retrospective cohort study discovered that the prevalence of PE in patients with COPD was 12.31 per 10,000 person-years, which was approximately fourfold higher than that in patients without COPD [6]. The prevalence of PE in patients with clinically diagnosed acute exacerbation of chronic obstructive pulmonary disease (AE-COPD) has been reported to range from 3.3% to 29.1% [7]. In recent years, the diagnosis of patients with suspected acute pulmonary embolism has greatly improved due to better clinical evaluation of the probability of PE, biomarkers, pretest probability, echocardiography, ventilation-perfusion (VQ) scan, and spiral computed tomography angiography (CTA) [8]. However, PE and COPD are very similar that the two are often indistinguishable clinically. PE is easily neglected in patients with COPD exacerbations because of their similar clinical characteristics, resulting in delayed treatment and poor prognosis [9]. Therefore, timely and effective evaluation has important clinical significance for suspected AE-COPD complicated with PE.

At present, the clinical diagnosis of PE mainly depends on spiral computed tomography pulmonary angiography (CTPA) [10]. However, for critically ill patients, CTPA examination can easily lead to missed diagnosis and untimely treatment, resulting in an increase in mortality. The prediction of PE seems to be an overwhelming task, especially in some community hospitals with limited resources. Thus, an accessible, cost-effective, and noninvasive clinical assessment method is needed to determine the possibility of COPD patients with PE. Recently, RDW increases observed in various diseases, including inflammatory bowel disease, cardiovascular disease (CVD), pulmonary disease (PD), and cerebrovascular accident, show that RDW can be an appropriate indicator for the differentiation of various diseases [11]. Therefore, in this study, our main purpose is to explore whether there is a correlation between RDW values and the development of PE in patients with COPD through a retrospective evaluation of COPD patients with PE.

## 2. Methods

### 2.1. Study Population

We retrospectively collected the date from January 2013 to December 2019 in Huai'an First People's Hospital. In this retrospective study, 125 patients were diagnosed with COPD. All patients were divided into a study group and a control group according to whether they also had PE. 40 patients with COPD complicated with PE were included in the study group and 85 patients in the control group. According to the location of PE, the study group was further divided into two: patients with unilateral PE and patients with bilateral PE.

The inclusion criteria were as follows: (1) patient age ≥ 45 years; (2) a COPD diagnosis defined according to the Global Initiative for Chronic Obstructive Lung Disease Report [12]. The diagnosis of COPD was confirmed by the pulmonary function test of airflow obstruction, with forced exhalation for one second/forced vital capacity < 70% that confirms the existence of persistent airflow limitation, and (3) the criteria set forth by the European Society of Cardiology Guidelines [13] regarding the diagnosis of PE. In a CTPA of the chest, one or more filling defects or obstructions were identified in the pulmonary artery (PA) or its branches, thus confirming the diagnosis of PE.

The exclusion criteria were as follows: (1) pregnant and lactating women, (2) patients with a history of blood transfusion in the past two weeks and hematological diseases (such as anemia, hematopoietic abnormalities, and hematologic malignancies), and (3) patients with incomplete or missing data.

The study protocol was approved by the ethics committee of Jiangsu Province Huai'an First People's Hospital and was carried out in accordance with the guidelines of the Helsinki Declaration. Due to the retrospective design of this study, the patient did not sign the informed consent form.

### 2.2. Data Collection

Age, sex, smoking index, COPD progression (years), body mass index (BMI), basic disease, and laboratory index were obtained by reviewing medical records on a computer. Laboratory parameters such as blood routine, arterial blood gas analysis, coagulation markers, and biochemical indications were also collected, and all data were obtained within 12 hours after admission to the hospital. CTPA results were classified as positive for PE when a filling defect was found in the PA. Blood routine index included white blood cell (WBC) count, red blood cell (RBC) count, hemoglobin (Hb), platelet (PLT) count, red cell distribution width (RDW), mean platelet volume (MPV), mean platelet volume (MPV), platelet distribution width (PDW), and hematocrit (HCT) (%).

### 2.3. Statistical Analysis

SPSS 26.0 (IBM Corp, Armonk, NY) was used for all statistical analyses. The normality of the data was tested by the Shapiro-Wilk method, and a value of *p* < 0.05 was considered a significant difference. The single variable comparisons of continuous variables used the two-sample independent *t*-test of normal distribution data or the nonparametric Mann–Whitney *U*-test of nonnormal distribution variables. The *χ*^2^ test was performed to compare categorical data. The numerical variables of the normal distribution were expressed as means ± standard deviations (SD), and the parameters of the nonnormal distribution were expressed by median-interquartile ranges (IQR). Categorical variables were expressed in frequencies and percentages. In univariate analysis, those parameters with a *p*  value < 0.05 were analyzed by binary logistic regression analysis with the “Enter” method. Logistic regression analysis was used to reveal the independent risk factors of PE in COPD patients. The discriminating performance of the RDW was evaluated using the receiver operating characteristic (ROC) curves. The corresponding areas under the curve (AUC) with 95% confidence intervals (CI) were used to compare the predictive probabilities. The optimal cutoff value was based upon the Youden index, and the cutoff values for RDW with sensitivity and specificity were calculated.

## 3. Results

### 3.1. Baseline Characteristics of the Study Population

This study retrospectively enrolled a total of 125 patients. 40 COPD patients with PE were included in the study group, and the remaining eighty-five patients were assigned to the control group. PE was located in the unilateral PA in 33 patients but was bilaterally found in 7 patients. Baseline demographic characteristics are shown in Table 1. There was no significant difference in average age and gender between the two groups (age: 71.63 ± 8.30 versus 70.31 ± 8.96, *p* = 0.434; male proportion: 80% versus 76.5%, *p* = 0.819). No significant difference was observed between the two groups with respect to BMI, smoking index, and course of the disease. History of diabetes, hypertension, and respiratory failure did not differ significantly between the study group and the control one.

### 3.2. Overall Comparison of Laboratory Parameters

No significant difference was observed among groups with respect to WBC, Hb, HCT (%), MCV, PDW, MPV, lymphocytes (%), mean corpuscular hemoglobin concentration (MCHC), platelet-large cell rate (P-LCR), monocyte (MONO) (%), cholesterol (CHOL), triglyceride (TG), creatinine (CREA), uric acid (UA), partial pressure of carbon dioxide (PCO_2_), potential of hydrogen (PH), activated partial thrombin time (TT), and thromboplastin time (APTT). The levels of RDW-SD and RDW-CV in the study group were significantly increased than those in the control group (47.5 versus 43.5, *p* < 0.001; 14.1 versus 13.1, *p* < 0.001). Compared with the control group, the albumin (ALB) of the study group decreased significantly (34.4 versus 37.7, *p* < 0.001). Patients with PE had a significantly higher median aspartate aminotransferase (AST) value (23 versus 13, *p* < 0.001), higher median AST value (19.5 versus 16, *p* < 0.001), and higher median lactate dehydrogenase (LDH-L) value (239.5 versus 175, *p* < 0.001) than the COPD group. The median (IQR) D-Dimer was 2.62 (1.87-9.25) in the COPD with the PE group and 0.4 (0.31-0.72) in the COPD without the PE group. D-Dimer was significantly higher in the study group compared to the control one (*p* < 0.001). The COPD with the PE group had significantly lower RBC, Hb, HCT, PLT, platelet crit (PCT) (%), eosinophil (EO) (%), lymphocyte (LYM) (%), oxygen saturation (SO_2_) (%), partial pressure of oxygen (PO_2_), and fibrinogen (FIB) and higher MPV, neutrophil (NEUT) (%), lactate (Lac), and prothrombin time (PT) than the control group.  Table 2 shows the blood routine, biochemical parameters, blood gas analysis, and coagulation indexes in COPD patients with and without PE.

### 3.3. Comparison of Biomarkers according to a Thrombus in Bilateral PA and Unilateral PA in Patients with PE

To evaluate the correlation between laboratory parameters and PE, we make a further comparison about the levels of RDW-SD, RDW-CV, and D-Dimer in patients categorized according to a thrombus in the bilateral PA and unilateral PA. The laboratory parameters are listed in Table 3. There was no significant difference in RDW-SD, RDW-CV, and D-Dimer between the two groups. LYM% in the bilateral group was significantly higher than that in the unilateral group (0.55 ± 0.29 vs. 0.92 ± 0.64, *p* = 0.019).

### 3.4. Logistic Regression Analysis of the Occurrence of PE

The parameters that were significantly different in univariate analysis were further included in the multivariate logistic regression model (*p* < 0.001). Independent risk factors for PE indicated by logistic regression model analysis are listed in Table 4. Multivariate analysis identified that LDH-L, RDW-SD, and D-Dimer were independent risk factors of PE in COPD patients, with odds ratios of 1.188 (95% CI, 1.048-1.348; *p* = 0.007), 1.012 (95% CI, 1.001–1.023; *p* = 0.032), and 1.516 (95% CI, 1.042–2.205; *p* = 0.030), respectively. Nevertheless, RDW-CV, ALB, AST, and alanine aminotransferase (ALT) did not remain as independent risk factors for PE.

### 3.5. Comparative Analysis of the Predictive Value of RDW and D-Dimer

**Figure 1:**
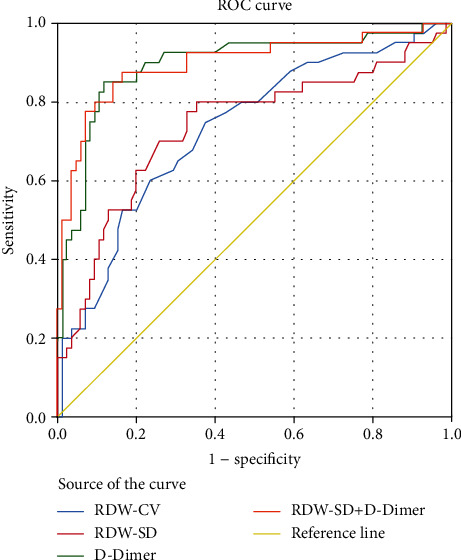
ROC curves for comparisons. ROC curves for determining the cutoff value of RDW-CV, RDW-SD, and D-Dimer for predicting PE in COPD patients. Abbreviations: RDW-SD: red blood cell distribution width standard deviation; RDW-CV: red blood cell distribution width coefficient of variation.

In order to predict PE in COPD patients, a receiver operating characteristic (ROC) curve was protracted. The detailed statistics of the predictive accuracy values of RDW and D-Dimer are listed in Table 5. The ROC curve analysis indicated that the AUC values for RDW-CV were 0.731; RDW-SD, 0.737; and D-Dimer, 0.895. The optimal cutoff values of RDW-SD, D-Dimer, and RDW-CV ratio for predicting PE were 44.55, 1.375, and 13.35, respectively, with sensitivity and specificity values of 80% and 64.7%, 85% and 88.2%, and 75% and 62.4%, respectively. The feasibility of the combined prediction of RDW-SD and D-Dimer was further tested to improve the diagnostic efficacy of PE secondary to COPD. The AUC value for RDW-SD combined with D-Dimer was 0.897, which was higher than that of simple RDW-SD or D-Dimer. The optimal cutoff value of RDW-SD+D-Dimer for predicting PE was 0.266, which generated a sensitivity of 87.5% and a specificity of 83.5%. The predictive parameters of PE in patients with COPD are shown in Figure 1.

## 4. Discussion

In the current study, RDW-CV and RDW-SD were significantly higher in COPD patients with PE than in COPD patients without PE. In addition, a higher RDW-SD might be associated with a significantly increased risk of PE than a lower RDW-SD. Furthermore, RDW-CV and RDW-SD can be used to predict PE in COPD patients. In these cases, the discriminative ability of RDW-SD combined with D-Dimer has been shown to be superior to RDW-SD or D-Dimer alone.

There are multiple mechanisms potentially linked to the increased risk of thromboembolism in COPD patients, including systemic inflammation, hypoxemia, enhanced oxidative stress, endothelial dysfunction, and a prothrombotic state [14]. Current studies have demonstrated RDW as a possible surrogate biomarker of inflammatory activity. Wang et al. stated that inflammation is a potential mechanism that can explain the relationship between RDW and chronic thromboembolic pulmonary hypertension [15]. This study showed that RDW-SD and RDW-CV were significantly higher in the study group than in the control one (47.5 versus 43.5, *p* < 0.001; 14.1 versus 13.1, *p* < 0.001). The mechanisms behind this conclusion have not been clearly explained. Ozgul et al. showed that RDW values were higher in the COPD group than in the controls [16]. This conclusion was recently supported by a study by Epstein et al., which suggested a number of explanations regarding the link between increased RDW during index exacerbation and higher readmission rates, which may be attributed to a variety of underlying metabolic abnormalities such as increased erythropoietin secretion and unrecognized acute worsening of cardiac function [17]. To date, the relationship between RDW and the occurrence of PE in COPD patients is a relatively uninvestigated area. RDW is associated with several inflammatory markers, such as acute-phase reactants C-reactive protein (CRP), erythrocyte sedimentation rate, and platelet count [18, 19]. Studies have shown that markers of systemic inflammation, such as CRP, IL-6, IL-8, and TNF-*α*, contribute to the development of thrombotic events [20]. We believe that ineffective erythropoiesis caused by chronic inflammation may contribute to an increase in the RDW level in COPD patients with PE. In addition, other pathogeneses that may be responsible for increased RDW in patients with COPD with poor prognosis after AE-COPD include increased oxidative stress, hypoxia, and poor nutritional status [21, 22].

A comprehensive review of diseases affecting the respiratory tract showed that the long-term response of RDW to hypoxia may be related to the strong response of endogenous erythropoietin to hypoxia, and hypoxemia (as SaO_2_) is correlated with RDW in PE and COPD [23]. However, this has not been extensively studied. Hypoxemia may be another reason for the increase in RDW levels in patients with COPD complicated with PE. In addition, oxidative stress has been considered to be an important determinant of RDW and is associated with cardiopulmonary thrombotic diseases [24].

In our study, thrombi were localized in the unilateral PA in 33 (82.5%) of the cases and in the bilateral PAs in 7 (17.5%) of the cases. Sunnetcioglu et al. conducted a study involving 148 patients with PE. It was reported that the thrombi were localized in the right PA in 56 cases (37.8%), left PA in 26 cases (17.5%), and bilateral PAs in 66 cases (44.5%) [25]. In addition, our study suggested that there was no significant difference in RDW-SD between unilateral thrombi and bilateral thrombi of PE.

Multivariable logistic regression analysis was used to evaluate the contribution of risk factors to COPD patients with PE. Our study showed that the prediction of PE by RDW-SD, LDH-L, and D-Dimer was not affected by other risk factors. A high D-Dimer level had a significantly increased risk of PE than a low D-Dimer level (OR 1.516, 95% CI 1.042-2.205). Taking into account the contribution of RDW-SD to the risk factors of PE, it was significantly associated with a 1.188-fold increase in the risk of PE (95% CI 1.048-1.348, *p* = 0.007). This indicates that early diagnosis of PE is very important for high-risk patients, and doctors could evaluate high-risk patients according to RDW-SD of COPD patients with PE.

As a relatively simple, inexpensive, and easily measurable laboratory factor, RDW has recently been shown to predict mortality and morbidity for many different diseases. Patel et al. used the mechanism model of RBC population dynamics in vivo for analysis and found that there are four possibilities for controlling RDW: (1) average volume reduction, (2) increase in the variance in increased reticulocyte volume, (3) increase in the heterogeneity of the erythrocyte volume reduction rate in peripheral blood circulation, and (4) delayed RBC clearance [26]. RDW is a powerful and independent predictor of morbidity in patients with cardiovascular events [27], advanced heart failure [28], and diabetes mellitus [29]. Some studies have shown that RDW is related to the severity of the disease, which may be helpful to predict the prognosis of PE. Ozsu et al. evaluated 702 patients with PE and found that RDW levels may provide a potential marker for mortality [30]. Zhou et al. discovered that blood RDW levels are a simple and practical indicator in predicting 30-day mortality in patients with PE [31]. Hammons et al. subsequently reported that there was a correlation between RDW and the prediction, severity, and survival of patients with acute PE [32]. Although it has been shown that RDW is associated with increased mortality in PE, few studies have focused on the ability of RDW to predict the possible occurrence of PE in COPD patients. In this study, the ROC curve analysis showed that the AUC of the D-Dimer for predicting PE was higher than that of RDW-SD, and the predictive ability of RDW-SD combined with D-Dimer was stronger than that of RDW-SD or D-Dimer. Hence, we believe that high RDW levels may be an indicator of PE in COPD patients.

## 5. Limitations of This Study

Our study has some limitations. First of all, the study was a single-center study; the sample size was relatively small, especially the COPD with the PE group. Secondly, this study was a retrospective study, and some critically ill patients were not allowed to undergo lung function tests for the sake of their safety when they were admitted. Third, RDW may be affected by iron and vitamin B12 levels [33], but this was not evaluated in our study. Finally, our study considered that inflammation may be one of the reasons for the increased RDW levels of PE in COPD patients, but the association between certain inflammatory factors (such as CRP and ESR) was not evaluated in our study.

## 6. Conclusion

In this study, we proved that COPD patients with PE have higher RDW-SD levels than COPD patients without PE. RDW-SD can be used as a potential biomarker for the diagnosis of PE in COPD patients, and the discriminative ability of RDW-SD combined with D-Dimer has been shown to be superior to RDW-SD or D-Dimer alone. RDW has broad application prospects in the prognosis of respiratory conditions, but their diagnostic role in COPD with PE patients is limited. Its role in prognosis requires a larger sample size and more multicenter and well-designed prospective clinical studies to confirm its applicability. In addition, further research is required to elucidate the underlying mechanism of the role of RDW in COPD patients with PE.

## Figures and Tables

**Table 1:** Baseline characteristics of participants with and without pulmonary embolism.

Characteristics	COPD with PE (*n* = 40)	COPD without PE (*n* = 85)	*p* value
Age, years (mean ± SD)	71.63 ± 8.30	70.31 ± 8.96	0.434
Gender (male) (*n*, %)	32 (80)	65 (76.5)	0.819
COPD progression (year)	0.204	0.204	0.204
BMI (kg/m^2^)	22.8 ± 3.44	23.30 ± 3.95	0.492
Smoking index (year root)	40 (0-600)	400 (0-800)	0.113
Respiratory failure (*n*, %)	16 (40.0)	25 (29.4)	0.307
Hypertension (*n*, %)	15 (37.5)	31 (36.5)	1.000
Diabetes (*n*, %)	7 (17.5)	11 (12.9)	0.587

Abbreviations: COPD: chronic obstructive pulmonary disease; PE: pulmonary embolism; BMI: body mass index.

**Table 2:** Comparison of the laboratory parameters between the two groups.

Parameters	COPD with PE (*n* = 40)	COPD without PE (*n* = 85)	*p* value
WBC (×10^9^/l)	8.37 (5.70-10.95)	8.18 (6.5-10.51)	0.979
RBC (×10^12^/l)	4.20 ± 0.74	4.49 ± 0.58	0.036
HB (g/l)	125.05 ± 17.88	134.54 ± 18.09	0.007
HCT (%)	37.7 (35.25-42.25)	41.2 (37.7-45.2)	0.015
MCV (fl)	93.27 ± 7.27	92.7 ± 4.95	0.611
MCH (Pg)	29.99 ± 2.41	30.02 ± 1.73	0.941
MCHC (g/l)	321.88 ± 15.88	323.85 ± 11.61	0.434
PLT (×10^9^/l)	163.5 (132.5-249)	211 (176-255)	0.013
RDW-SD (fl)	47.5 (44.8-51.95)	43.5 (41.8-45.8)	*p* < 0.001
RDW-CV (%)	14.1 (13.35-15.15)	13.1 (12.6-13.8)	*p* < 0.001
PDW (fl)	12.25 (11, 13.9)	11.5 (9.9-13.2)	0.050
MPV (fl)	10.43 ± 1.3	9.94 ± 1.16	0.047
PCT (%)	0.18 (0.13-0.27)	0.21 (0.18-0.25)	0.049
P-LCR (%)	28.68 ± 9.38	25.59 ± 8	0.059
EO (×10^9^/l)	0.03 (0.01-0.09)	0.08 (0.02-0.19)	0.001
NEUT (%)	79.3 (75.15-85.1)	75 (68.3-82.3)	0.016
LYM (%)	12.55 (8.25-15.55)	15.7 (10.1-21.1)	0.032
MONO (%)	6.4 (4.9-8.3)	6.8 (5.4-8.2)	0.670
EO (%)	0.75 (0-1.25)	1.2 (0.3-2.4)	0.012
ALB (g/l)	34.4 (32.7-37.7)	37.7 (34.9-40.5)	*p* < 0.001
ALT (*μ*/l)	23 (12-32.5)	13 (8-18)	*p* < 0.001
AST (*μ*/l)	19.5 (15.5-31.5)	16 (13-19)	*p* < 0.001
LDH-L (*μ*/l)	239.5 (195.5-278.5)	175 (154-201)	*p* < 0.001
CHOL (mmol/l)	4.31 (3.4-4.64)	4.1 (3.6-5)	0.610
TG (mmol/l)	1.23 (0.85-1.45)	0.96 (0.78-1.28)	0.084
CREA (*μ*mol/l)	59.4 (52-86.05)	65 (54-74)	0.914
UA (*μ*mol/l)	239.1 (173-373.05)	248 (210-322.3)	0.899
PH	7.45 (7.4-7.46)	7.42 (7.4-7.45)	0.267
SO_2_ (%)	93.5 (88.65-96.45)	95.7 (93.4-98.5)	0.004
PO_2_ (mmHg)	64.25 (52.8-77.1)	73.2 (63.3-73.2)	0.007
PCO_2_ (mmHg)	42.2 (35.8-52.4)	43.4 (40-50.1)	0.209
Lac (mmol/l)	1.75 (1.35-2.45)	1.5 (1.2-1.9)	0.034
PT (sec)	13.7 (13.1, 15.3)	13.3 (12.7, 13.9)	0.007
APTT (sec)	36.7 (32.2-40.8)	36.1 (33.9-39.7)	0.801
TT (sec)	16.9 (15.65-19.54)	16.7 (15.8-17.6)	0.307
FIB (g/l)	3.76 (2.21-5.06)	4.63 (3.6-5.92)	0.002
D-Dimer (*μ*g/ml)	2.62 (1.87-9.25)	0.4 (0.31-0.72)	*p* < 0.001

Abbreviations: COPD: chronic obstructive pulmonary disease; PE: pulmonary embolism; WBC: white blood cell; RBC: red blood cell; HB: hemoglobin; HCT: hematocrit; MCV: mean corpuscular volume; MCH: mean corpuscular hemoglobin; MCHC: mean corpuscular hemoglobin concentration; PLT: platelet; RDW-SD: red blood cell distribution width standard deviation; RDW-CV: red blood cell distribution width coefficient of variation; PDW: platelet distribution width; MPV: mean platelet volume; PCT: platelet crit; P-LCR: platelet-large cell rate; EO: eosinophil; NEUT: neutrophil; LYM: lymphocyte; MONO: monocyte; ALB: albumin; ALT: alanine aminotransferase; AST: aspartate aminotransferase; LDH-L: lactate dehydrogenase; CHOL: cholesterol; TG: triglyceride; CREA: creatinine; UA: uric acid; PH: potential of hydrogen; SO_2_: oxygen saturation; PO_2_: partial pressure of oxygen; PCO_2_: partial pressure of carbon dioxide; Lac: lactate; PT: prothrombin time; APTT: activated partial thromboplastin time; TT: thrombin time; FIB: fibrinogen.

**Table 3:** Laboratory parameters based on the location of pulmonary embolism.

	Unilateral thrombus (*n* = 33)	Bilateral thrombus (*n* = 7)	*p*
RBC (×10^12^/l)	4.21 ± 0.77	4.18 ± 0.64	0.938
HB (g/l)	125.73 ± 18.67	121.86 ± 14.35	0.609
HCT (%)	39.03 ± 6.06	38.39 ± 4.73	0.794
PLT (×10^9^/l)	165 (128.5, 264)	151 (136, 253)	0.873
RDW-SD (fl)	47.6 (44.8, 52.4)	46 (44.8, 51.6)	0.593
RDW-CV (%)	14.1 (13.4, 15.2)	13.6 (13, 16.1)	0.569
PCT (%)	0.2 (0.1, 0.3)	0.2 (0.1, 0.3)	0.901
EO (×10^9^/l)	6.5 (3.8, 8.5)	7.9 (7.1, 15.5)	0.088
NEUT (%)	0.9 (0.6, 1.5)	1.5 (0.6, 1.9)	0.319
LYM (%)	0.55 ± 0.29	0.92 ± 0.64	0.019
EO (%)	79.1 (74, 85.4)	79.5 (78.4, 85)	0.776
ALB (g/l)	34.58 ± 4.1	36.67 ± 3.02	0.212
ALT (*μ*/l)	23 (12, 31)	16 (10, 55)	0.943
AST (*μ*/l)	19 (15, 32.5)	20 (16, 31)	0.762
LDH-L (*μ*/l)	230 (191.5, 289)	273 (218, 275)	0.569
SO_2_ (%)	93.8 (88.7, 96.5)	90.2 (87.8, 98.5)	0.510
PO_2_ (mmHg)	64.5 (56.9, 77.1)	56.4 (51.9, 98.2)	0.488
Lac (mmol/l)	29.9 ± 7.23	30.71 ± 8.74	0.795
PT (sec)	1.8 (1.4, 2.5)	1.4 (1.2, 3.4)	0.761
Fib (g/l)	3.76 ± 1.91	3.56 ± 1.28	0.790
D-Dimer (*μ*g/ml)	2.5 (1.7, 15.4)	4.4 (2, 4.9)	0.656

Abbreviations: RBC: red blood cell; Hb: hemoglobin; HCT: hematocrit; PLT: platelet; RDW-SD: red blood cell distribution width standard deviation; RDW-CV: red blood cell distribution width coefficient of variation; PCT: platelet crit; P-LCR: platelet-large cell rate; EO: eosinophil; NEUT: neutrophil; LYM: lymphocyte; ALB: albumin; ALT: alanine aminotransferase; AST: aspartate aminotransferase; LDH-L: lactate dehydrogenase; SO_2_: oxygen saturation; PO_2_: partial pressure of oxygen; Lac: lactate; PT: prothrombin time; FIB: fibrinogen.

**Table 4:** Independent risk factors for PE indicated by logistic regression model analysis.

Variable	Odds ratio	95% CI	*p* value
RDW-SD (fl)	1.188	1.048-1.348	0.007
RDW-CV (%)	1.013	0.858-1.197	0.877
ALB (g/l)	0.903	0.785-1.039	0.155
ALT (*μ*/l)	1.008	0.975-1.042	0.639
AST (*μ*/l)	0.985	0.902-1.075	0.733
LDH-L (*μ*/l)	1.012	1.001-1.023	0.032
D-Dimer (*μ*g/ml)	1.516	1.042-2.205	0.030

Abbreviations: RDW-SD: red blood cell distribution width standard deviation; RDW-CV: red blood cell distribution width coefficient of variation; ALB: albumin; ALT: alanine aminotransferase; AST: aspartate aminotransferase; LDH-L: lactate dehydrogenase.

**Table 5:** Comparison of the discriminative ability of some parameters to predict PE.

Parameters	RDW-CV	RDW-SD	D-Dimer	RDW-SD+D-Dimer
Cutoff	13.350	44.550	1.375	0.266
AUC	0.731	0.737	0.895	0.897
95% CI	0.636-0.827	0.635-0.839	0.828-0.962	0.829-0.966
Sensitivity (%)	75.0	80.0	85.0	87.5
Specificity (%)	62.4	64.7	88.2	83.5

Abbreviations: AUC: area under the curve; CI: confidence interval; RDW-SD: red blood cell distribution width standard deviation; RDW-CV: red blood cell distribution width coefficient of variation.

## Data Availability

The data used to support the findings of this study are included within the supplementary information file (available here).

## Supplementary Materials

Supplementary Materials (1) In the gender column, 1 is for male and 2 is for female; (2) in the respiratory failure, hypertension, and diabetes columns, 0 is for no and 1 is for yes; (3) in the location of PE columns, 0 is for no and 1 is for yes; (4) BMI is defined as a person's weight in kilograms divided by the square of the height in meters (kg/m^2^); (5) smoking index is defined as the average number of cigarettes per day multiplied by the number of years of smoking; (6) abbreviations: WBC: white blood cell; RBC: red blood cell; HB: hemoglobin; HCT: hematocrit; MCV: mean corpuscular volume; MCH: mean corpuscular hemoglobin; MCHC: mean corpuscular hemoglobin concentration; PLT: platelet; RDW-SD: red blood cell distribution width standard deviation; RDW-CV: red blood cell distribution width coefficient of variation; PDW: platelet distribution width; MPV: mean platelet volume; PCT: platelet crit; P-LCR: platelet-large cell rate; EO: eosinophil; NEUT: neutrophil; LYM: lymphocyte; MONO: monocyte; PH: potential of hydrogen; SO_2_: oxygen saturation; PO_2_: partial pressure of oxygen; PCO_2_: partial pressure of carbon dioxide; Lac: lactate; PT: prothrombin time; APTT: activated partial thromboplastin time; TT: thrombin time; FIB: fibrinogen; ALB: albumin; ALT: alanine aminotransferase; AST: aspartate aminotransferase; LDH-L: lactate dehydrogenase; CHOL: cholesterol; TG: triglyceride; CREA: creatinine; UA: uric acid.
