# Supplementary material for: Predictive Value of Red Blood Cell Distribution Width in Chronic Obstructive Pulmonary Disease Patients with Pulmonary Embolism
Source: Anal Cell Pathol (Amst). 2020 Jul 21;2020:1935742. doi: 10.1155/2020/1935742 (PMC7391120; doi:10.1155/2020/1935742)
Supplement: Supplementary Materials — (1) In the gender column, 1 is for male and 2 is for female; (2) in the respiratory failure, hypertension, and diabetes columns, 0 is for no and 1 is for yes; (3) in the location of PE columns, 0 is for no and 1 is for yes; (4) BMI is defined as a person's weight in kilograms divided by the square of the height in meters (kg/m2); (5) smoking index is defined as the average number of cigarettes per day multiplied by the number of years of smoking; (6) abbreviations: WBC: white blood cell; RBC: red blood cell; HB: hemoglobin; HCT: hematocrit; MCV: mean corpuscular volume; MCH: mean corpuscular hemoglobin; MCHC: mean corpuscular hemoglobin concentration; PLT: platelet; RDW-SD: red blood cell distribution width standard deviation; RDW-CV: red blood cell distribution width coefficient of variation; PDW: platelet distribution width; MPV: mean platelet volume; PCT: platelet crit; P-LCR: platelet-large cell rate; EO: eosinophil; NEUT: neutrophil; LYM: lymphocyte; MONO: monocyte; PH: potential of hydrogen; SO2: oxygen saturation; PO2: partial pressure of oxygen; PCO2: partial pressure of carbon dioxide; Lac: lactate; PT: prothrombin time; APTT: activated partial thromboplastin time; TT: thrombin time; FIB: fibrinogen; ALB: albumin; ALT: alanine aminotransferase; AST: aspartate aminotransferase; LDH-L: lactate dehydrogenase; CHOL: cholesterol; TG: triglyceride; CREA: creatinine; UA: uric acid. [file 1935742.f1.docx]

In this retrospective study, 125 patients were diagnosed with COPD. According to the results of CTPA, patients were divided into COPD with PE group (n = 40) and COPD without PE group (n = 85). According to the location of PE, the first group was further divided into two: patients with unilateral PE and patients with bilateral PE. All these data were listed in the supplementary documents, including: (1) in the gender column, 1 is for male and 2 is for female; (2) in the Respiratory failure, Hypertension, and Diabetes columns, 0 is for no and 1 is for yes; (3) in the location of PE columns, 0 is for no and 1 is for yes; (4) BMI is defined as a person’s weight in kilograms divided by the square of the height in meters (kg/m2); (5) Smoking index is defined as the average number of cigarettes per day multiplied by the number of years of smoking; (6) abbreviations: WBC-[white blood cell](file:///C:/Users/13297/AppData/Local/youdao/dict/Application/8.8.1.0/resultui/html/index.html#/javascript:;); RBC-red blood cell; HB-[hemoglobin](file:///C:/Users/13297/AppData/Local/youdao/dict/Application/8.8.1.0/resultui/html/index.html#/javascript:;); HCT-hematocrit; MCV-mean corpuscular volume; MCH-mean corpuscular hemoglobin; MCHC-[mean corpusular hemoglobin concentration](file:///C:/Users/13297/AppData/Local/youdao/dict/Application/8.8.1.0/resultui/html/index.html#/javascript:;); PLT-platelet; RDW-SD-red blood cell distribution width standard deviation; RDW-CV-red blood cell distribution width coefficient of variation; PDW-platelet distribution width; MPV-mean platelet volume; PCT-platelet crit; P-LCR-platelet-large cell rate; EO-eosinophil; NEUT-neutrophil; LYM-lymphocyte; MONO-monocyte; PH-potential of hydrogen; SO2-oxygen saturation; PO2-partial pressure of oxygen; PCO2-partial pressure of carbon dioxide; Lac-lactate; PT-prothrombin time; APTT-activated partial thromboplastin time; TT-thrombin time; FIB-fibrinogen; ALB-albumin; ALT-alanine aminotransferase; AST-aspartate aminotransferase; LDH-L-lactate dehydrogenase; CHOL-cholesterol; TG-triglyceride; CREA-creatinine; UA-uric acid.

BMI is defined as a person’s weight in kilograms divided by the square of the height in meters (kg/m^2^). The smoking index was defined as the average root number per day multiplied by years of smoking.

Patients diagnosed with AECOPD (n = 185) were registered in this retrospective study. All patients evaluated for PH in our study underwent Doppler echocardiography and were divided into study and control groups depending on whether they also had PH. 101 AECOPD patients with PH were included in the PAH group, and the remaining eighty-four patients were assigned to the COPD group. Clinical characteristics and baseline laboratory tests (routine blood test (RBT), blood gas analysis, and amino terminal pro-B-type natriuretic peptide (NT-proBNP)) were tested at enrollment. All these data were listed in the supplementary file. There are some things particularly revelatory here: (1) in the gender column, 1 is for male and 2 is for female; (2) in the Respiratory failure, Hypertension, and Diabetes columns, 1 is for no and 2 is for yes; (3) BMI is defined as a person’s weight in kilograms divided by the square of the height in meters (kg/m2); (4) the definition of the smoking index is the average root number per day multiplied by years of smoking; (5) inflammatory indices were calculated as follows: NLR = neutrophil counts/lymphocyte counts; PLR = platelet counts/lymphocyte counts; SII = platelet counts × neutrophil counts /lymphocyte counts; (6) abbreviations: LAD—left atrium diameter; LVDD—left ventricular end diastolic diameter; RAD—right atrium diameter; RVD—right ventricular diameter; PTRV—peak tricuspid regurgitation velocity; AECOPD—acute exacerbation of chronic obstructive pulmonary disease; PH—pulmonary hypertension; BMI—-body mass index; WBC—white blood cell; RBC—red blood cell; NLR—neutrophil-to-lymphocyte ratio; PLR—plateletto- lymphocyte ratio; SII—systemic-immune-inflammation index; PaCO2—partial pressure of carbon dioxide; HCO3-—- bicarbonate ion; Lac—lactic acid; PASP—pulmonary arterialsystolic pressure; PaCO2—partial pressure of carbon dioxide;

HCO3

-—bicarbonate ion; NLR—neutrophil-to-lymphocyte

ratio; PLR—platelet-to-lymphocyte ratio; SII—systemicimmune-

inflammation index. (Supplementary Materials)
